# Albinism research in a Southern African setting: unique findings

**DOI:** 10.1007/s12687-025-00786-3

**Published:** 2025-03-26

**Authors:** Jennifer G. R. Kromberg, Robyn A. Kerr

**Affiliations:** Division of Human Genetics, School of Pathology, Faculty of Health Sciences, University of the Witwatersrand and National Health Laboratory Service, PO Box 1038, Johannesburg, 2000 South Africa

**Keywords:** Albinism, South Africa, Clinical, Epidemiology, Psychosocial, Genetics

## Abstract

Research on oculocutaneous albinism (OCA) in the black African population has been ongoing for 52 years (1971–2023) in the Division of Human Genetics, University of the Witwatersrand, Johannesburg, South Africa. The aim of the present study was to review all the relevant published articles and focus on selected articles with unique findings. The results showed that unique findings were reported in psychosocial, cultural, epidemiological, clinical and molecular fields of study. The local prevalence of albinism was found to be 1 in 3900, higher than that reported in many other countries, although a worldwide review on prevalence showed that only 26/193 (13%) countries had published figures; the commonest types of OCA found were OCA2 and then OCA3; the high rate of skin cancer was documented; and the natural history of OCA described. Molecular studies showed that the 2.7 kb deletion mutation in the *OCA2* gene is the common mutation in OCA2 locally, and further identified unique mutations in *TYRP1* causing rufous albinism (OCA3) in this population. An early study found that after the birth of a child with OCA maternal-infant bonding was delayed, and only established some months later. Further research revealed that superstitions and myths surrounded the birth and the death of a person with OCA, and the belief that powerful medicines could be made from body parts, was very disturbing. Genetic causes of OCA were poorly understood by affected individuals, their relatives and communities, and genetic counselling is essential. In summary, over 30 studies were undertaken and published over a period of five decades, and many presented unique findings on this under-researched inherited condition.

## Introduction

Albinism is a well-recognised disorder which, although it occurs in about 1 in 5000 individuals in Africa (Hong et al. 2006), is a rare and under-researched condition in most parts of the world. Since the early 1900s it has been understood to be genetic with a recessive mode of inheritance (Davenport and Davenport 1910). It is caused by biallelic gene mutations, at a single gene locus, which result in a lack of melanin pigment production in the cell (Rooryck et al. 2009).

There are two main types of albinism: oculocutaneous albinism (OCA) and ocular albinism (OA). In the first case the affected individual has depigmentation of both eyes and skin, in the second case only the eyes lack pigment. OCA is far more common than OA, particularly in Africa, where very few cases of OA have been reported and there is a higher prevalence of OCA than on any other continent. This article will focus on OCA, and on Southern Africa where much of the African albinism research, which has resulted in several unique findings, has been undertaken (Kromberg and Manga 2018).

In OCA, lack of melanin in the eye results in low vision, and lack of melanin in the skin results in a predisposition to skin cancer (squamous and basal cell). There are 7 types of OCA and these have been differentiated (Monteliu et al. 2014). Further, Manga (2018) has published a comprehensive discussion on the molecular and biochemical basis of OCA, which includes a detailed description of OCA 1–7. OCA type 1 (MIM: # 203100) results from variation at the *TYR* locus which leads to a lack of tyrosinase, an enzyme essential during melanin biosynthesis. This is the most severe form of albinism with complete lack of pigment in the skin, hair and eyes; individuals have whitish skin and hair and light blue eyes. OCA type 2 (MIM: # 203200) results from variation at the *OCA2* locus (previously referred to as the *P* locus), and individuals have a slightly milder phenotype with an accumulation of some red/yellow phaeomelanin as the individual ages, and hazel-coloured eyes. OCA type 3 (# 203290) is significantly less severe than types 1 and 2 and is referred to as rufous or red oculocutaneous albinism (ROCA), and in individuals of African descent the phenotype is one of a coppery brown skin, lighter than normally pigmented individuals but not ‘white’ as for OCA1 and OCA2 in this population. In OCA3 secondary changes in the retinas can be observed but the visual impairment is minimal. The other types of OCA are rare worldwide and have not been described in Africa.

In the early 1970s a research project on albinism was initiated by a team (a medical doctor, medical social worker, and later, a nurse) working at the South African Institute for Medical Research (SAIMR, now the National Health Laboratory Service, NHLS) and University of the Witwatersrand, in the Division of Human Genetics, in Johannesburg, South Africa; this research continued for five decades. The project focussed on the epidemiology of albinism initially, since individuals with the condition were often observed but the prevalence was unknown. The study was expanded into the psychosocial field and later into the clinical, and molecular aspects of the condition, as the necessary expertise became available in the department. Research was supported by long-term funding from the South African Medical Research Council (for two sessions of five years each), and thereafter by various smaller research grants.

The purpose of this review paper is to outline some of the unique findings resulting from the many studies on OCA performed over the last 52 years (1971–2023), in this setting. These findings produced unique insights into the albinism condition at the time they were undertaken, since they were focussed on albinism in Southern Africa (understudied in this setting, despite a relatively high prevalence). Some of the studies made a unique contribution to world knowledge, others were more specifically unique to Africa and /or Southern Africa. The article will not take a chronological approach (which might have shown how research methodologies and research expertise developed over the decades) to the presentation of the many research projects undertaken. Instead, it will cover the epidemiological and clinical research topics first, followed by the genetic and molecular topics and, lastly, the psychosocial and cultural studies. Finally, for the sake of completeness, the human rights issues that have arisen while this research has been undertaken, but have not yet been studied in detail, will be briefly discussed.

## Clinical and medical aspects

### Epidemiology of albinism

One of the first projects on albinism to be initiated (in 1971) involved the local epidemiology of albinism, and this was followed 30 years later (in 2021) with a systematic review on the global prevalence data on albinism. Both these studies were unique: the first had never been undertaken previously in Africa and the second had never been undertaken but allowed for the assessment of the availability of data worldwide.

The frequency of albinism in southern Africa was determined in a Johannesburg-based project (Kromberg and Jenkins 1982). The study was carried out on the population of Soweto (a large Johannesburg satellite suburb, where the great majority of the black population, from many different ethnic groups, lived at the time) which then numbered about 803,000. Multiple methods of ascertainment were employed, including visits to schools, community clinics, local hospital clinics and word of mouth from one family to another. All the individuals with OCA (*N* = 213) were interviewed in their homes by the medical social worker (JGRK) and the nurse in the team and, where they gave consent, they were enrolled into the study. At that time a pharmaceutical company was approached to donate a large supply of sun-barrier cream, and this was bottled and supplied free of charge to all affected individuals (in whom it’s daily use is essential to prevent the development of skin cancer, in the sunny climate of Southern Africa).

The results of this study showed that 1 in 3900 individuals in this community had OCA. On phenotype examination, all individuals had OCA type 2, characterised by the white skin but yellowish hair and hazel-coloured eyes. The local population in this setting was unique since it was made up of about 10 different ethnic groups, the largest of which were the Tswana and Zulu groups. The Tswana group was found to have a prevalence of 1 in 3481, while the Zulu group had a lower frequency of 1 in 4459. When the prevailing cultural factors were investigated and compared, the consanguineous marriage rate was found to differ significantly and the Tswana group had a higher rate (42%) as compared to the Zulu group (4.3%, among whom marriage to a close relative is taboo). This finding partly explained the difference in prevalence observed in these two local groups (Kromberg and Jenkins 1982).

Further studies on OCA prevalence were carried out in two of South Africa’s neighbouring countries, Swaziland (now Eswatini) and Botswana, which had never been studied previously. The Swazi study was situated in the Hhohho district with its population of 160,000 people. Altogether 54 individuals with OCA in 39 families were identified and 49 individuals were interviewed. On examination most (44/49) had OCA2, but OCA3 was diagnosed clinically in five children. The consanguinity rate was 17%, and 58% of families had a family history. The final prevalence figure was 1 in 1951, higher than in most other Southern African countries (Kromberg 2018c). In Botswana, a large, isolated village, with 18,300 people, was investigated. There were 14 individuals with OCA in nine families living there. The prevalence was 1 in 1307 and the carrier rate 1 in 19. Again, on examination, most affected individuals had OCA2, but OCA3 was identified in three siblings with an unusual reddish yellow skin colour and fair hair (Kromberg et al. 2012).

An older smaller study, undertaken by researchers working in the field of cancer epidemiology, estimated that in one ethnic group, the Xhosa, living in rural Transkei (now Eastern Cape province), 1 in 3759 people had albinism (Oettle 1963). Later, Rose (1974) working in the same area, found that 1 in 3000 individuals had OCA. These figures, although only related to one ethnic group, were in-keeping with those found in the Kromberg and Jenkins study (1982) described above.

Several populations worldwide have been described with exceptionally high prevalence rates. These are all small, isolated populations with high rates of albinism due to founder effect and the frequency of consanguineous matings in the community. Examples include the Zuni (1 in 247) and Hopi (1 in 227) Indians in North America (Witkop et al. 1972; Woolf et al. 1965) and in Africa, population isolates in Botswana (1 in 1307), Zimbabwe (1 in 1000) and Nigeria (1 in 1100) (Kromberg 2018c; Lund et al. 1997; Okoro 1975). Also, in certain groups, a selective advantage of carriers has been suggested - Woolf and Dukepoo (1969) describe a form of ‘cultural’ selection amongst the Hopi Indians whereby males with OCA did not have to work in the fields as a protective measure against sun exposure, but remained in the village, giving them a slight sexual advantage. African studies have suggested that carrier females, having a lighter skin colour, were preferred as marriage partners, contributing to an increased frequency of OCA-related genetic variants in the population (Kromberg 1987; Oettle 1963).

### Worldwide prevalence of OCA2

At the suggestion of a representative from the United Nations, working for human rights for persons with albinism, members of the South African research team were encouraged, more recently, to undertake a systematic review of the literature, interrogating the existing prevalence data for OCA worldwide. The use of data, including prevalence data, is essential for decision-making within health care systems and for informing health policy. The systematic review (Kromberg et al. 2023) aimed to clarify what data were available regarding worldwide disease epidemiology, identify under- and over-estimates of frequency in specific populations, and ultimately determine whether a generalizable, worldwide figure could be proposed. Results showed that only a few countries worldwide (26/193; 13%) had produced prevalence figures for OCA. By continent, African studies were disproportionately represented (13/32; 41%). The highest prevalence rates were reported in population isolates, such as those in Arizona (Witkop et al. 1972). Low prevalence rates reported for populations in Europe are probably inaccurate as the phenotype is not always striking in fair skinned individuals. Most of the data were outdated, and there were very few good studies with large, clearly defined population samples. However, the findings showed that it was not possible to determine a single, generalizable worldwide prevalence for OCA, and highlighted the importance of generating good and current data in order to create public awareness and advocate for better healthcare, especially in underserved populations.

### Clinical characteristics

Due to the lack of melanin and pigmentation in individuals with OCA, the skin is highly sensitive to the ultraviolet rays of the sun. In 1952 Cohen et al., working in the Transvaal province (now the Gauteng province) of South Africa, documented the high risk of skin cancer in individuals with OCA. Sun damage can occur very early in life, unless preventive and protective care is taken. To assess skin damage occurring in the local population, 111 individuals with OCA were studied (Kromberg et al. 1989). Among those aged 1–19 years 20% (15/76) already had solar damage, and this frequency increased with age. The sites most often affected were the face, cheeks, and eyelids. None of those aged 50 + years were free of skin cancer and the commonest type was squamous carcinoma, which was generally aggressive and difficult to treat successfully. This study provided unique information on the nature of the age-related skin damage associated with OCA and motivated the recommendation that skin-barrier creams should be used from an early age onwards.

As discussed in Williams (2018) visual problems occur in all individuals with OCA due to the lack of melanin, which is required for the correct routing of the optic tract in the development of the embryo. Most affected individuals have nystagmus, photophobia, refractive errors and myopia, which require regular visual assessment, from an early age, and treatment (Kammer 2018). Individuals with OCA have abnormal decussation of the optic fibres in the visual pathway, which results in a reduction in binocular and stereoscopic vision (Williams 2018). In order to address the question of whether heterozygote carriers of OCA2 had an optic tract defect similar to that found in those with OCA, a study was carried out locally and visual evoked potentials (VEP) testing was performed on 15 obligatory carriers (Castle et al. 1988). The results showed that these carriers had symmetry on monocular pattern stimulation and therefore had normal decussation at the chiasm, indicating that VEP testing is not necessary for carriers.

### Types of OCA in South Africa

As the long-term study progressed it was recognised that not all individuals with OCA had OCA2 and some had different physical characteristics, suggestive of rufous albinism (now known as OCA3). In order to provide information on this group of individuals with albinism, a study was initiated in the late 1980s and a cohort of individuals with OCA3 were examined in Johannesburg and Lesotho (Kromberg et al. 1990). This group of individuals was found to have significantly different colouring to that of persons with OCA2. The skin colour of individuals with OCA3 was reddish and hair colour yellow to gold, eyes were usually brownish, photophobia was mild, and eyesight only slightly affected. A follow up study, in which the common types of OCA (OCA2, with a subtype, and OCA3) found in Southern Africa were described in detail, was undertaken (Kromberg et al. 2012). A unique and useful table differentiating these three types of OCA was compiled and included in this publication.

### Natural history

In black African populations OCA can usually be diagnosed at birth, since the infant will have white hair and skin, in striking contrast to the (usually) normally pigmented parents. This is in contrast to white European populations where the signs in a newborn are not obvious immediately and the condition might only become apparent later, when the poor vision is detected, and the diagnosis is often delayed in this population group (Healey et al. 2014).

During the course of the various studies on albinism the opportunity arose to observe and document the natural history of the condition in many individuals of different ages (Kromberg 2018d). In black African families the signs of albinism at birth are the infant’s white skin and hair colour which are usually obvious to the attending health professionals as well as the parents. When the baby’s eyes open the light eye colouring, atypical red reflex, nystagmus, photophobia and lack of focus generally become apparent. Many of those present at the birth express their shock and surprise at the child’s appearance, which is so visibly different to that of the black mother (Kromberg et al. 1987).

Information on children with OCA was collected and reported recently (Kromberg et al. 2020). In the first year of life the child requires ophthalmology and dermatology assessments, and the mother needs to learn to care for the child’s special needs. Sun damage can begin early in life and daily application of sun barrier-cream (SPF 50) is essential and life-long, especially in equatorial and tropical countries. As the children grow, they need to learn to avoid sunshine, wear hats at all times, as well as long sleeved cotton clothing. However, they also need to recognise the stigmatisation and marginalisation that they might often be exposed to, and learn to deal with it (Kromberg et al. 2020). Once schooling (preferably in a community school) starts the teacher should be informed of the child’s limited vision, and the need for a seat in the front of the class, away from the glare, and wearing a hat, if necessary (Kammer 2018). Also, large print texts, visual aids (e.g., a monocular and/or magnifier) and extra time for tests should be considered. The children should be encouraged to achieve intellectually (since intelligence is in the normal range), so that they are eligible for indoor employment, instead of outdoor jobs where their skin is at risk of sun damage.

Marriage can be problematic for individuals with OCA2 in Africa as, although acceptance in the community is increasing, it may stop short of marriage (Kromberg and Jenkins 1984). At this life stage individuals with OCA should seek genetic counselling themselves so that they can understand the cause of their condition and the risk of recurrence (Kromberg 2018b). Affected individuals need to know that they are unlikely to have children with OCA (unless they happen to marry someone who is a carrier of the same type of OCA), but that all their children would be obligate carriers. Throughout their lives they need to have regular annual skin and vision clinical checks, in order to optimally manage their visual difficulties, prevent sun damage, and treat any lesions that occur early (Hartshorne and Manga 2018). A useful Guidelines article, on the Management of Albinism, has been published by a French team (Moreno-Artero et al. 2021).

With age, individuals with OCA usually develop solar keratoses and skin wrinkling, but if their general care and health is good, then lifespan should be within the normal range. The death myth should be discredited so that it does not worry affected individuals and they should be empowered to explain it away as pure superstition (Kromberg 1992).

## Genetics and molecular aspects

### The development of molecular genetics research

Research on the molecular causes of albinism started in the middle 1980s, when a molecular genetics laboratory was set up in the Division. The research focused on OCA2, since clinical examinations (and early hair bulb tests, used to identify the presence or absence of tyrosinase) of many individuals with OCA from the local black population showed that this was the commonest type occurring in South Africa. At this time the gene for OCA2 had not been identified, and linkage studies were undertaken as a first step. These initially only excluded large parts of the genome (Colman et al. 1993; Kromberg et al. 1990), but linkage to chromosome 15q was ultimately established (Kedda et al. 1994; Ramsay et al. 1992). The gene that, when mutated, causes OCA2 was identified by researchers in the USA (Rinchik et al. 1993) and was named the *P* gene, after the pink-eyed dilute mouse in which it was first identified. Durham-Pierre et al. (1994), investigating a community of mixed racial origin in the USA, described a mutation in the *P* gene (now called the *OCA2* gene), a 2.7 kb intragenic deletion, and postulated that this mutation was of African origin. Stevens et al. (1995), working in the Division, then showed that this mutation was the common OCA2 mutation in the southern African population and accounted for 78% of OCA2 chromosomes. On testing of several other populations, including those from Zambia, Cameroon, Zaire and the Central African Republic, it was found that the 2.7 kb deletion occurs in other sub-Saharan countries as well, but with reduced frequency (Stevens et al. 1997). This wide distribution of the 2.7 kb deletion confirmed the African origin of this mutation. Affected individuals who had been found to carry either one or two non-2.7 kb deletion alleles were later investigated (Kerr et al. 2000). Only a few (4/52) pathogenic mutations were discovered, and no other common mutation was identified, among non-2.7 kb alleles in individuals with albinism from the different regions of sub-Saharan Africa investigated in this study (South Africa, Lesotho, Zimbabwe, and the Central African Republic).

The local molecular research work was expanded in the early 1990s to include individuals with the brown (BOCA, *N* = 10) and rufous (ROCA, *N* = 19) types of albinism who had been identified during field trips and other clinical work (Kromberg et al. 1990). The gene associated with ROCA was mapped and found to be the *TYRP1* gene on chromosome 9p, and two common mutations were identified that together accounted for 95% of disease-causing mutations (Manga et al. 1997). As this was the third locus that had been shown to cause an OCA phenotype in humans, ROCA was labelled OCA3. Brown albinism was also investigated and found to map to the OCA2 locus on chromosome 15q; it was thus shown to be a sub-type of OCA2, presenting with a slightly milder phenotype as compared to classic OCA2 (Manga et al. 2001). In the small cohort of BOCA individuals investigated in this study, 9/10 individuals were heterozygous for the 2.7 kb deletion and the second mutation remained unknown.

### Genetic testing and prenatal diagnosis

In Southern Africa, testing for the common 2.7 kb mutation can confirm the presence of the mutation and resolve a queried clinical diagnosis, detect whether an at-risk person is a carrier, and can be used for prenatal testing. Genetic testing for the 2.7 kb deletion mutation was set up in 1999 at the NHLS in Johannesburg. In a small study, the findings from 101 DNA tests which were performed between 1999 and 2016 were analysed (Kerr and Kromberg 2018). The results showed that most of the testing was carried out on black African patients (66/101), the remaining individuals being white, mixed ancestry, Malagasy or Indian. Altogether, 50 of the tests were diagnostic tests, 43 were carrier tests and seven were prenatal tests. Only black individuals were found to be homozygous for the 2.7 kb mutation, and, as expected, no white nor Indian patients had this mutation. Among the carrier tests for at-risk individuals, 23/43 were confirmed to be carrying the mutation. On prenatal diagnosis (7 cases), one foetus was found to be homozygous for the mutation (the mother decided to continue the pregnancy and gave birth to a healthy infant with albinism). Prenatal diagnosis for OCA is still a contentious issue and very few tests have been performed locally as these are rarely requested by at-risk families (Kromberg et al. 2015).

## Psychosocial and cultural issues

### Intellectual maturity and body image

The first unique project to be undertaken in the series of studies on psychosocial issues (initiated in 1971), was on intellectual maturity, rather than intelligence quotient (IQ) (since no valid culture-free testing tools were available to test IQ in this population at the time) and body image in young people with OCA (Manganyi et al. 1974). The Draw a Person culture-free test (Harris 1963; Witkin et al. 1962) was used and participants had to draw three figures, a male, female and self-portrait. Altogether 28 young individuals (mean age 11.9 years, range 7–16 years) with OCA, together with 28 matched unaffected controls from the local black population in Johannesburg, were studied. The results indicated that the level of intellectual maturity was slightly higher in the participants with OCA than in the controls (significant at the 2% level) and well within the normal range. The findings on body image showed that the subjects with OCA had slightly more differentiated image characteristics than did the controls, but that body image could be an artefact of the intellectual maturity dimension. However, the data also showed that the participants with OCA had a more negative self-evaluation and identity than the controls. The researchers concluded that albinism must be a complicated mode of being in society and the state of persons with OCA must be one of marginality. These findings presented unique data on albinism in Africa which had never been published previously. However, in one older study intellectual abilities in African American individuals with OCA, living in the USA, were investigated, and no intellectual deficit was found when they were compared with their siblings without OCA (Beckham 1946). Later, Stewart and Keeler (1965) studied intelligence in San Blas Indians with OCA and a control group and found no statistical difference between the two groups.

### Adjustment to having albinism

Adjustment can be considered as “*a condition of harmonious relation to the environment wherein one is able to obtain satisfaction for most of one’s needs and to meet fairly well the demands physical and social put upon one” (*English and English 1970, p13). In order to assess adjustment in individuals with OCA an exploratory psychosocial project was undertaken. Participants included 35 young urban black South Africans with OCA (mean age 17 years) and 35 matched unaffected controls, living in the urban environment of Soweto, Johannesburg (Kromberg and Jenkins 1984). These young people were interviewed using a schedule of items covering adjustment and attitudes, drawn from Bell’s Adjustment Inventory and the Science Associates Youth Inventory (1956), and the experience of one of us (JGRK) (this schedule was tested in a pilot study with 50 participants and modified accordingly). Results showed that those with OCA appeared to be as well-adjusted as the controls. However, the former group described more physical and psychosomatic problems than the latter. Psychosomatic symptoms can indicate unexpressed anxiety and depression (Vernon 1964), and this may be due to persons with OCA having to live in a partially stigmatising and rejecting society (in which they are often referred to in the local language as ‘nkau’ or monkey). When the female (*N* = 14) participants’ responses were examined, they showed that this sub-group were significantly better adjusted than their matched controls. This finding is possibly due to the females with OCA giving responses that showed a better self-concept (in comparison with the controls), and also to the fact that the control females showed a higher level of acceptance of albinism than did the control males. The small samples in this study did not allow for any more detailed statistical analysis, so the results should be seen as trends only. Further research is required to better understand the complex psychology associated with albinism.

### The response of the parents to the birth of an infant with albinism

The maternal response to the birth of an infant with OCA was investigated in an in-depth research study (Kromberg et al. 1987). This project was also carried out in Soweto, Johannesburg, and its neighbouring tertiary state hospital (Chris Hani Baragwanath Academic Hospital, CHBAH) where most local mothers went for labour and delivery. The participants were mothers (*N* = 37) with an infant with OCA and the matched controls (*N* = 37) were mothers with a baby with a black skin colour. These dyads were followed for 12 months, the mothers were interviewed, and maternal-infant interaction was observed (and scored with a checklist), at three-month intervals, starting soon after delivery. The findings showed significant differences between the experimental and control groups. Most mothers with a child with OCA were shocked and upset at the time of delivery (the few exceptions were generally those mothers who were aware of a family history of the condition). After delivery they had some difficulty identifying with their child, resulting in some emotional detachment, withdrawal and indifference. However, by three months most of these mothers showed increased interaction and bonding with their child and by nine months most expressed decreased feelings of unhappiness. There were observable and significant differences in the behaviour of the mothers with a child with albinism compared to the control mothers initially, but not at three months after the birth. However, the experimental mothers could only verbalise their reduced unhappiness and acceptance of the child at nine months after the birth. These findings suggest that acceptance on the behavioural level might occur sooner than acceptance at the cognitive level. Learning to cope may take longer to achieve in thought as opposed to in action, a phenomenon also observed with regard to infants with Down syndrome in a study by Cunningham (1982). This unique study confirms that recovery from the effects of having a child with a disability (which includes albinism) is a gradual process and takes time (Kennedy 1970) and there may be some long-lasting chronic sorrow (Kromberg et al. 1987).

A small exploratory study, on the few available fathers (*N* = 10/37, who were partners of ten participating mothers, and volunteered to participate) of an infant with OCA, was included in this project (Kromberg 2018a). Initially, mothers (*N* = 37) reported that fathers were also upset and shocked when they first saw their child, a few denied paternity and/or deserted mother and child. The ten fathers who participated were only interviewed once (at different stages after the birth), and some described delayed acceptance and later attachment to the child. This finding was only a trend as the sample was too small (and a biased and self-selected one) to draw any conclusions and a full study on paternal attitudes and responses to the birth of a child with albinism is required.

### Cultural issues, myths and superstitions

Early on in these studies, as the research team worked in the community and got to know severalpeople with OCA and their families, it became obvious that many myths and superstitions surrounded the disorder in local black communities. Such myths are often associated with rare conditions, and, in some cultures the myths might become an integral part of the worldview which is “*essentially a (cognitive) attempt to make sense of the world and to impose meaning on it”* (Hammond-Tooke 1989, p33). Since people often do not understand why rare disorders occur, they may allocate their own ideas, or those of their ancestors, regarding aetiology (Baker et al. 2010). Research showed that causes were often believed to be related to the mother’s behaviour during pregnancy (e.g., laughing or scoffing at individuals with OCA, getting too close to them, or becoming infected due to proximity), her wrongdoing, ignoring taboos and/or a traditional healer’s curse (Kromberg et al. 1987). Another common myth surrounds the death of individuals with OCA, as they are believed not to die natural deaths but rather to “disappear” mysteriously at the end of their lives.

This death myth was investigated as part of the Kromberg and Jenkins (1984) study on 35 young people with OCA and 35 matched controls. About 43% of the control group believed the myth, supporting their view by adding that they had never seen a person with OCA die, nor the corpse of a person with OCA, so they wondered why that was so. About 31% of the participants with OCA were unsure about what would happen at the end of their lives, which made them uncertain of their humanity and mortality (suggesting life was unstable, insecure, and difficult for them). Only 37% responded confidently that individuals with OCA died like other people did. A few (reflecting their own uncertainty) asked the interviewer if the myth was true. One control individual stated: “I always say albinos are never buried, they walk away and die far from home”. Another said: “They fall ill, recover quickly and after this disappear to die away from their people. Whether death follows is still a mystery” (Kromberg 1992).

This unusual myth is believed in other African countries, including Nigeria (Imafidon 2019), and individuals with OCA have been reported as not ‘real’ people (since ‘real’ people in Africa are ‘black’ (see Imafidon 2019), but rather as spirits or ghosts. To support this argument, it has been reported that when black people first saw white people in Africa, they said they were not human and attributed spiritual powers to them (Mutwa 1966). Also, one research participant stated (about people with albinism) that “being black by birth but not by colour may be one of their major problems; this deprives them of the companionship of other black children” (Kromberg and Jenkins 1984, p104). Another study (Phatoli et al. 2015) further explored beliefs and practices around albinism in Africa and presents the psychosocial challenges of “being black in a white skin”.

Another more sinister myth is also widely believed in several African countries, and that holds that medicine made from the body parts of individuals with OCA is very powerful. Belief in this myth has resulted in attacks on individuals with OCA, in order to gain bodily tissue, often involving the severing of a limb, and in some instances, murder. Traditional healers then make medicine, using the tissue, which can be sold at a high price. Those who buy it range from politicians and businessmen to gold miners and fishermen, as they are often seeking more success in life. Attempts are being made in several central African countries to debunk and counteract this myth (Clarke and Beale 2018; Mostert and Weich 2017).

### Genetic counselling for albinism

Genetic counselling was made available to the general public in Johannesburg in 1972 (Jenkins et al. 1973). Thereafter weekly genetic clinics were set up in all the tertiary hospitals in the city. A research project on albinism and genetic counselling issues found that the participants only had a very poor understanding of the causes and genetics of albinism (Kromberg and Jenkins 1984; reworked in Kromberg 2018b). The outcome of this study was that guidelines for genetic counselling for families with a member with OCA were drawn up and should include:

1. A clear description of albinism and the medical facts.
2. An explanation of the genetic cause of albinism and risks of recurrence.
3. A discussion of the clinical complications, i.e., skin lesions and poor vision (as well as the necessary health monitoring and timely treatment to minimise the side-effects), the prognosis, and how best to manage the condition.
4. A discussion of the myths (e.g., the death myth and myth regarding making medicine from body parts) and superstitions.
5. Discussion regarding widespread negative attitudes (including negative attitudes towards marriage) and stigmatisation, as well as community attitudes and misconceptions in general.
6. Consideration of the schooling and employment issues and referral for vocational guidance.

Some years later, in 2015, the uptake of the counselling service in the CHBAH genetics clinic was audited (Kromberg et al. 2015). The findings from this project showed that 38 families with a child with OCA were counselled in a 4 year-period and they had benefitted from the knowledge they gained. Continued uptake of the service was good and 9/12 (75%) of the expected families used the service annually. This good uptake was likely the outcome of the Division’s active local long-term research programme that had alerted health professionals to the need for genetic counselling and motivated them to refer families to the service.

## Other issues

### Albinism and human rights

Although no local research project has been undertaken as yet on the topic of human rights and albinism, no discussion of the condition and its surrounding issues would be complete without a mention of the topic. The relationship between albinism, disability and human rights (legal, health, educational and social rights) is complex (Clarke and Beale 2018) and this issue has been considered and discussed in lectures and tutorials in the Division. Although the human rights predicament of people with albinism varies around the world, there is widespread stigmatisation. In Africa, the nature and severity of human rights abuses require immediate attention and resources (Clarke and Beale 2018). The basic human rights of respect for life and the dignity of the individual, the right to liberty and security, to equal access to health and education services, to community participation, and to freedom of choice, are all too often ignored and neglected in the case of individuals with albinism. They are often marginalised, not treated as real people (Imafidon 2019), seen as socially unacceptable, misunderstood and structurally vulnerable, which constrains their ability to access healthcare and pursue a healthy lifestyle (Reimer-Kirkham et al. 2019). To rectify the human rights violations the following is needed by people with OCA: Counselling and support from knowledgeable health professionals, from birth onward; access to support groups; regular healthcare (specifically for vision and skin care); advocacy and education. Further, ongoing relevant community education, with the aim of increasing understanding and reducing the stigmatisation, is also essential (Kromberg et al. 2023).

## Conclusion

In conclusion, the results from the five decades of research work focused on albinism in South Africa, undertaken by the staff of the Division of Human Genetics, University of the Witwatersrand, have produced many unique findings described in over 24 original research papers, two reviews and a further nine book chapters. A monograph entitled “Albinism in Africa” was also written and published (Kromberg and Manga 2018). Findings in the field of the social sciences, as well as those in the clinical, medical and molecular fields have been described. The major findings from selected articles highlighted and summarised above, include the following: prevalence of OCA is higher as compared to non-African populations; a review of the literature showed figures reported for the prevalence of albinism worldwide are mostly outdated and somewhat inaccurate; the rate of skin cancer on the face and head is concerning; the two common types found locally are OCA2 and then OCA3; the 2.7 kb mutation in the *OCA2* gene is the mutation causing 78% of OCA2 in South Africa; the unique mutations causing OCA3 are identified; intelligence is within the normal range; maternal-infant bonding is often delayed; superstitions and myths abound; genetic counselling services are essential; the violation of human rights of people with albinism is still problematic.

Much knowledge has been gained from this long-term research project. Further studies, such as those on quality of life, are required in order to better understand the complicated mode of being in society as experienced by individuals with OCA. Genetic studies can further elucidate the molecular causes of the various types of OCA found in Southern Africa. Expanded community education is required if people with OCA are to be better integrated into, and accepted by, their communities.

Statements and Declarations.

## Data Availability

No datasets were generated or analysed during the current study.
